# Derivation of Patient-Defined Adverse Cardiovascular and Noncardiovascular Events Through a Modified Delphi Process

**DOI:** 10.1001/jamanetworkopen.2020.32095

**Published:** 2021-01-04

**Authors:** Louise Y. Sun, Jillian Rodger, Lisa Duffett, Heather Tulloch, Andrew M. Crean, Aun-Yeong Chong, Fraser D. Rubens, Erika MacPhee, Thierry G. Mesana, Douglas S. Lee, Sean van Diepen, Rob S. Beanlands, Marc Ruel, Ann-Marie Julien, Jean Bilodeau

**Affiliations:** 1 Division of Cardiac Anesthesiology, University of Ottawa Heart Institute, Ottawa, Ontario, Canada; 2 University of Ottawa School of Epidemiology and Public Health, Ontario, Canada; 3 Institute for Clinical Evaluative Sciences, Ontario, Canada; 4 Department of Medicine, University of Ottawa, Ottawa, Ontario, Canada; 5 Division of Prevention and Rehabilitation, University of Ottawa Heart Institute, Ontario, Canada; 6 Division of Cardiology, University of Ottawa Heart Institute, Ottawa, Ontario, Canada; 7 Division of Cardiac Surgery, University of Ottawa Heart Institute, Ontario, Canada; 8 Clinical Operations, University of Ottawa Heart Institute, Ontario, Canada; 9 Peter Munk Cardiac Center, University Health Network, Toronto, Ontario, Canada; 10 Department of Critical Care and Division of Cardiology, Department of Medicine, University of Alberta, Edmonton, Alberta, Canada; 11 University of Ottawa Heart Institute Patient Alumni Association, Ottawa, Ontario, Canada

## Abstract

**Importance:**

There is little evidence to support patient-centered outcomes in patients with cardiovascular disease.

**Objective:**

To derive patient-defined adverse cardiovascular and noncardiovascular events (PACE) through a consensus-based process.

**Design, Setting, and Participants:**

This pan-Canadian, consensus-based, qualitative study used an iterative Delphi method to achieve consensus within a 35-member panel consisting of patients with cardiovascular diseases and their caregivers and clinicians. The process included 4 rounds of online questionnaires, followed by an in-person final consensus meeting. Data analysis was performed in September 2019.

**Main Outcomes and Measures:**

Defining PACE as a 5-item composite outcome.

**Results:**

Thirty-five potential panelists consented to participate, including 11 clinicians (8 men [73%]) and 24 patients and caregivers (13 men [54%]). Twenty-nine (83%), 28 (80%), 26 (74%), and 23 (66%) of the panelists participated in each of respective the online rounds. A shortlist of 11 patient-defined items was further refined at the in-person meeting, which 20 of the panelists attended. The PACE definition that was decided through the consensus process was a composite of severe stroke necessitating hospitalization for 14 days or longer or inpatient rehabilitation, ventilator dependence, new onset or worsening heart failure, nursing home admission, or new onset dialysis.

**Conclusions and Relevance:**

This study defined PACE as a versatile, patient-centered outcome through a consensus process with input from patients, caregivers, and clinicians. Given the paucity of patient-centered outcomes in cardiovascular research, PACE may be considered as a potential outcome after methodological evaluation of its reliability.

## Key Points

**Question:**

Which adverse cardiovascular and noncardiovascular events are most relevant to patients?

**Findings:**

In this qualitative study of patients with advanced cardiovascular diseases and their caregivers and clinicians, a consensus-based definition of patient-defined adverse cardiovascular and noncardiovascular events (PACE) was reached using a modified Delphi process; the definition included severe stroke necessitating hospitalization for 14 days or more or inpatient rehabilitation, ventilator dependence, new onset or worsening heart failure, nursing home admission, and new onset dialysis.

**Meaning:**

Given the paucity of patient-centered outcomes in cardiovascular research, the concept of PACE may be applied in future epidemiological and intervention studies to ensure that management of cardiovascular disease is founded on outcomes that are important and relevant to patients, caregivers, and clinicians.

## Teaser

This qualitative study attempts to derive patient-defined adverse cardiovascular and noncardiovascular events through a modified Delphi consensus-based process involving panel discussions with patients and their caregivers and clinicians.

## Introduction

Therapeutic decisions are challenging for patients with advanced cardiovascular disease, because there is a paucity of evidence to support patient-centered outcomes in this group.^1,2^ Traditional cardiovascular trials have sometimes been termed *tombstone trials*^3^ because of their focus on major adverse cardiac events and death. However, a prior survey^2^ of cardiovascular patients found that important outcomes identified by patients were very different from those used in traditional trials. Because patients with advanced cardiovascular disease are likely to be elderly with substantial comorbidities, they are more often concerned with how treatment might impact their ability to lead an independent life with less impairment and outside of chronic care institutions. Our group previously explored the concept of disability-free survival as a patient-defined outcome after coronary revascularization.^2^ We conducted the present study to refine this outcome by integrating the perspectives of patients and their caregivers, with input from expert clinicians. We term this outcome patient-defined adverse cardiovascular and noncardiovascular events (PACE).

## Methods

### Modified Delphi Technique

The 5-round, modified consensus process took place between April 2019 and September 2019 and consisted of a 4-round web-based Delphi questionnaire in accordance with guidelines,^4,5^ followed by an in-person consensus meeting. The Delphi method is a consensus-based, iterative process that uses repetitive questionnaires to gather information from a selected panel. It is widely used in health research to develop consensus statements and quality indicators through serial surveys or rounds.^6^

Approval was obtained from the research ethics board of the University of Ottawa Heart Institute (UOHI). Agreement to participate in the Delphi process was taken as implied consent. The reporting of this study follows the Consolidated Criteria for Reporting Qualitative Research (COREQ) reporting guideline.^7^

### Selection of Panelists

We recruited a 35-member panel representing patients with cardiovascular diseases, along with caregivers and clinical experts who routinely participate in their care. The patients and caregivers were recruited through the UOHI Patient Alumni Association via open email invitations through the alumni listserv and posting on the official alumni website, on the basis of having a confirmed diagnosis of cardiovascular disease treated with medical therapy and/or cardiac surgery and/or catheter-based procedure, left ventricular assist device implantation, or heart transplantation. Clinical expertise was defined by the possession of theoretical knowledge and practical experience in making treatment decisions for patients with advanced cardiovascular diseases. The clinical panel was chosen to represent diversity in terms of clinical specialty and practitioner gender, experience, and geography. It consisted of 3 cardiac surgeons, 1 cardiac anesthesiologist, 1 cardiac surgical intensivist, 1 interventional and 3 noninterventional cardiologists, 1 clinical health psychologist, and 1 registered nurse specializing in the care of cardiac patients. All clinicians, with the exception of 1 community-based intensive care physician, accepted the invitation to participate. There was diverse ethnic and geographical representation among panel members. Ethnicity was self-defined by panel members. The intended participants received an email containing a summary of the background, objective, design, and time needed for the study.

### Questionnaires

We have previously derived disability-free survival as a patient-centered outcome by surveying more than 3000 patients with cardiovascular disease.^2^ This definition served as the basis for the first questionnaire. Expanding on this patient-derived composite of stroke, nursing home admission, and recurrent, nonelective hospitalizations of more than 3 episodes per year, we solicited input from nonpanelist patients and clinical experts to add new items of potential impact (eTable 1 in the Supplement). Because our objective was to generate PACE outcomes that are readily linked with administrative data, we replaced conditions such as immobility and inability to perform self-care with more measurable surrogates, such as paraplegia and nursing home admission. The online questionnaire was pilot-tested by 2 patients and 2 health care professionals who were not members of the panel.

The questionnaires were administered anonymously using SurveyMonkey over 4 rounds (eTables 1, 2, 3, and 4 in the Supplement). The link and instructions for each of the questionnaires were provided to the panelists by email. At each round, the panelists were asked to rank items in order of perceived contribution to disability and provide free text to suggest additional relevant conditions to be considered for ranking during the next round.

Between rounds, data were summarized by 2 independent researchers (L.Y.S. and J.R.) who were not a part of the panel. Refinements to the questionnaire were discussed within the core group (L.Y.S., J.R., and L.D.), and the most highly ranked items along with new free text items were synthesized into the next round. We aimed to be inclusive during the first round and removed only items for which fewer than 10% of the panelists had voted as being important. For subsequent rounds, items that were deemed as important by fewer than 25% of the panelists were considered to be nonconsensus and removed. No financial compensation was offered, and participation could be withdrawn at any time.

### In-Person Delphi Panel Meeting

The fifth and final consensus-based round was conducted in person. Only those who participated in the online rounds were invited to the in-person meeting. A total of 20 participants were first divided into 4 small groups, each composed of 2 clinicians, 3 patients and/or caregivers, and a trained facilitator to engage the group in informed, patient-centered discussions to achieve consensus on a short list of 5 priority items. The patients were encouraged to drive the discussion, while the clinicians served as subject experts to provide clinically relevant perspectives to ensure that patients and caregivers properly understood each of the disability-related items and their clinical and quality of life implications. Once 5-item lists were achieved within the small groups, all participants rejoined the large group and engaged in further consensus-based discussions to condense the 4 sets of priority items into a final shortlist of 5 items.

### Statistical Analysis

Data were visualized in SurveyMonkey. During the in-person meeting, consensus was recorded manually. Data analysis was performed in September 2019.

## Results

Thirty-five potential panelists consented to participate. The characteristics of panel members are summarized in Table 1. Of these participants, 11 were clinicians (8 men [73%]; median [interquartile range], 18 [10-22] years of independent practice experience) and 24 were patients and caregivers (13 [54%]). The 5-round Delphi process is summarized in the Figure.

**Table 1.** Characteristics of Clinician, Patient, and Caregiver Panelists

Characteristic	Panelists, No. (%)
Clinicians (n = 11)	
Demographic characteristics	
Male	8 (73)
Race/ethnicity	
White	8 (73)
Asian	2 (18)
Indigenous	0
Other	1 (9)
Duration of independent practice, median (interquartile range), y	18 (10-22)
Specialty	
Cardiac	
Surgeon	3 (27)
Anesthesiologist	1 (9)
Surgical intensivist	1 (9)
Cardiologist	
Interventional	1 (9)
Noninterventional	3 (27)
Clinical psychologist	1 (9)
Registered nurse	1 (9)
Provinces of Canada	
Ontario	10 (91)
Alberta	1 (9)
Patients and caregivers (n = 24)	
Demographic characteristics	
Male	13 (54)
Race/ethnicity	
White	16 (67)
Asian	5 (21)
Indigenous	1 (4)
Other	2 (8)
Patient	21 (88)
Provinces of Canada	
Ontario	22 (92)
Quebec	1 (4)
Newfoundland	1 (4)
Therapeutic intervention for ischemic cardiomyopathy	
Coronary artery bypass grafting	8 (33)
Percutaneous coronary intervention	18 (75)
Left ventricular assist device	1 (4)
Heart transplant	1 (4)
Medical therapy only	3 (12)

**Figure.**
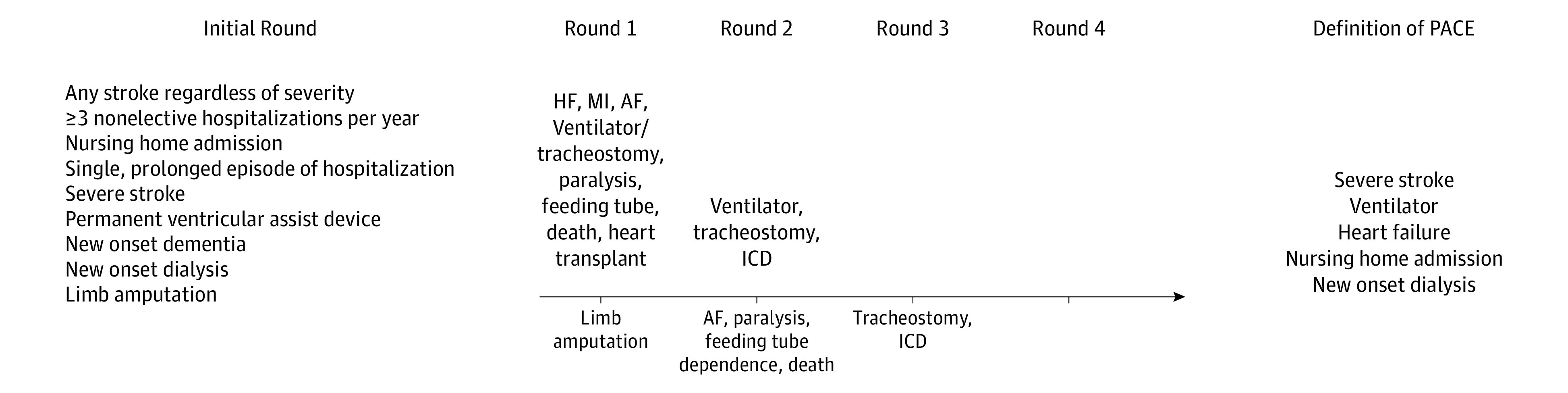
Summary of the 5-Round Delphi Consensus Process to Arrive at the Final Definition of Patient-Defined Adverse Cardiovascular and Noncardiovascular Events (PACE) Text above the arrow represents outcomes added, and text below the arrow represents outcomes that were removed before entering the next round. AF indicates atrial fibrillation; HF, heart failure; ICD, implantable cardioverter defibrillator; and MI, myocardial infarction.

### Questionnaire Round 1

Twenty-nine panelists (83%) responded to round 1, of whom 8 were clinicians, 17 were patients, and 4 were caregivers (Table 2). The round 1 questionnaire is presented in eTable 1 in the Supplement. There was nonconsensus on limb amputation, which was excluded from the subsequent round. The rest of the items from the round 1 questionnaire were added to round 2 along with new items recommended by the respondents. These new items included new or worsening heart failure requiring hospitalization, myocardial infarction, atrial fibrillation, tracheostomy, ventilator dependence, immobility or paralysis, dependence on feeding tube, and death.

**Table 2.** Role of Participants in Each Survey Round and Panel

Role	Participants, No. (%)				
	Round 1 (n = 29)	Round 2 (n = 28)	Round 3 (n = 26)	Round 4 (n = 23)	Round 5 (n = 20)
Patient	17 (59)	19 (68)	17 (65)	15 (65)	9 (45)
Caregiver	4 (14)	3 (11)	4 (15)	3 (13)	3 (15)
Clinician	8 (27)	6 (21)	5 (20)	5 (22)	8 (40)

### Questionnaire Round 2

In round 2, 28 (80%) of those who consented responded. Of the respondents, 6 were clinicians, 19 were patients, and 3 were caregivers (Table 2). The round 2 questionnaire is presented in eTable 2 in the Supplement. Consensus was reached on most items, with the exception of atrial fibrillation, immobility or paralysis, dependence on feeding tube, and death. The consensus items, along with implantable cardioverter defibrillator per recommendation by respondents, were added to round 3. Also, at the recommendation of respondents, we combined heart transplant and left ventricular assist device as a single item, and split tracheostomy and ventilator dependence into 2 separate items for round 3.

### Questionnaire Rounds 3 and 4

A total of 26 (74%) of those who consented responded to round 3 and 23 (66%) to round 4 (Table 2). Consensus was reached on most items during round 3 (eTable 3 in the Supplement) with the exception of tracheostomy and implantable cardioverter defibrillator, and no new items were added by respondents. The primary purpose of round 4 was to provide context for the in-person meeting. Therefore, all 11 items were retained after this round, and rankings provided by panelists were used to initiate the in-person discussions.

### In-Person Delphi Panel Meeting

A total of 20 panelists participated in the in-person meeting. Of these panelists, 9 were patients, 3 were caregivers, and 8 were clinicians (Table 2). During this meeting, the top 4 to 5 consensus items from each of the small groups (Table 3) were discussed within the overall group and condensed into a final shortlist of 5 items (Box). This definition of PACE consisted of severe stroke (necessitating hospitalization for ≥14 days or inpatient rehabilitation), ventilator dependence, heart failure requiring hospitalization, nursing home admission, and new onset dialysis.

**Table 3.** Priority Items as Defined by Each of the Small Groups at the In-Person Meeting, Listed in Order of Importance

Group 1	Group 2	Group 3	Group 4
Severe stroke	Ventilator dependence	New onset dementia	Nursing home admission
Ventilator dependence	New onset dementia	Severe stroke	New onset dialysis
Heart failure requiring hospitalization	Severe stroke	Ventilator dependence	Hospitalization ≥40 d
Nursing home admission	Heart failure requiring hospitalization	Hospitalization ≥40 d	Heart transplant or ventricular assist device
New onset dialysis	Heart transplant or ventricular assist device	Not applicable	Not applicable

Box. **Components of Patient-Defined Adverse Cardiovascular and Noncardiovascular Events (PACE)**

Severe stroke
Ventilator dependence
Heart failure requiring hospitalization
Nursing home admission
New onset dialysis

## Discussion

We conducted a 5-round Delphi consensus study of patients with cardiovascular disease, their clinicians, and caregivers, to collaboratively cocreate PACE as a patient-defined outcome. This unique process improves the clinical and social relevance of the outcome measure by integrating the goals and perspectives of those who provide and receive care.

### PACE as a Patient-Defined Outcome

A previous version of the patient-defined outcome disability-free survival was derived from a single patient survey without input from caregivers and clinical experts.^2^ This outcome was a composite of stroke of any severity, long-term care admission, and nonelective hospitalizations of 3 or more episodes per year. Through iteratively soliciting the perspectives of a representative panel that also includes caregivers and clinicians, we updated this outcome to include severe stroke, which is more likely to result in a lasting disability, taking into account that some instances of stroke end in recovery with minimal functional impairment. Other components, such as nursing home admission, ventilator dependence, new onset or worsening heart failure, and new onset dialysis, all reflect patients’ unwillingness to accept severe restrictions on personal mobility, freedom, and independence.

Patients with advanced cardiovascular disease are particularly prone to experiencing procedural failure, complications, morbidity, and worsening impairment after hospitalization.^8^ It is, therefore, not surprising that these patients are more often concerned about how treatments might affect their personal freedom and mobility, and the burden their illness would place on family and caregivers, rather than longevity alone.^3,9,10,11,12^ Treatment decisions have a very real consequence on many aspects of a patient’s life. A patient’s ability to make informed decisions is also influenced by the emotional and logistical repercussions of being diagnosed with the disease, together with limitations in health literacy. The establishment of PACE fulfills a critical need in the care of cardiovascular patients, by incorporating patient perspectives and patient-derived data into the design of clinical studies.^13,14,15^ PACE was founded on the principle that patient engagement will improve uptake of the evidence and make the decision-making process more relevant and empowering for patients.^10,16^

Given the paucity of patient-centered outcomes in cardiovascular medicine, research to establish the prevalence, risk factors, and mitigation strategies for PACE will help to advance the field. PACE provides potential domains for a measure of patient-centered outcomes, which could be used in the context of population-based epidemiological studies and prospective randomized trials of intervention effectiveness in patients with cardiovascular diseases.

### Limitations

This study has several limitations. First, by design, our results are influenced by participant views. However, the national panel consisted of a diverse group of medical experts, patients, and caregivers. The Delphi design allowed patients to freely express their needs and expectations, while preserving the context of the discussion through clinician input to ensure that key items were well understood by all participants. Second, the attendance of patient panelists was much lower for the in-person round than the online rounds. This likely reflects challenges with mobility and/or inability to travel long distances. With the advent of digital communication technology, video conferencing could be considered in future studies to optimize attendance in a patient-centered manner. Third, to enable linkage with administrative data, we used surrogates such as paralysis and nursing home admission for loss of personal independence. However, the versatility of PACE will also enable population-based, practice-changing research to truly bring patients back in the center of the care they receive. Fourth, PACE was derived in the context of the Canadian health care system. Future research should consider the applicability of the Delphi process in various regions throughout the world.

## Conclusions

This qualitative study derived PACE as a versatile patient-centered outcome through a consensus process with input from patients, caregivers, and clinicians. Given the paucity of patient-centered outcomes in cardiovascular research, PACE may be applied in future epidemiological and intervention studies to ensure that management of cardiovascular disease is founded on outcomes that are important and relevant to patients, caregivers, and clinicians.

## Notes

Supplement. **eTable 1.** Description of Patient Centered Outcome Results: Questionnaire Round 1 **eTable 2.** Description of Patient Centered Outcome Results: Questionnaire Round 2 **eTable 3.** Description of Patient Centered Outcome Results: Questionnaire Round 3 **eTable 4.** Description of Patient Centered Outcome Results: Questionnaire Round 4
