# Supplementary material for: Derivation of Patient-Defined Adverse Cardiovascular and Noncardiovascular Events Through a Modified Delphi Process
Source: JAMA Netw Open. 2021 Jan 4;4(1):e2032095. doi: 10.1001/jamanetworkopen.2020.32095 (PMC7783543; doi:10.1001/jamanetworkopen.2020.32095)
Supplement: Supplement. — eTable 1. Description of Patient Centered Outcome Results: Questionnaire Round 1 eTable 2. Description of Patient Centered Outcome Results: Questionnaire Round 2 eTable 3. Description of Patient Centered Outcome Results: Questionnaire Round 3 eTable 4. Description of Patient Centered Outcome Results: Questionnaire Round 4 [file jamanetwopen-e2032095-s001.pdf]

Supplemental Online Content

Sun LY, Rodger J, Duffett L, et al. Derivation of patient-defined adverse cardiovascular and noncardiovascular events through a modified Delphi process. *JAMA Netw Open*. 2021;4(1):e2032095. doi:10.1001/jamanetworkopen.2020.32095

eTable 1. Description of Patient Centered Outcome Results: Questionnaire Round 1
eTable 2. Description of Patient Centered Outcome Results: Questionnaire Round 2
eTable 3. Description of Patient Centered Outcome Results: Questionnaire Round 3
eTable 4. Description of Patient Centered Outcome Results: Questionnaire Round 4

This supplemental material has been provided by the authors to give readers additional information about their work.

© 2021 Sun LY et al. *JAMA Network Open.*

eTable 1. Description of Patient Centered Outcome Results: Questionnaire Round 1

Outcome	Value given to outcome	Included in next iteration (Y/N)
Any stroke regardless of severity	6	Y
≥3 non-elective hospitalizations per year	4	Y
Nursing home admission	4	Y
Single prolonged episode of hospitalization (≥40 days)	5	Y
Severe stroke (hospitalization ≥14 days or needing rehabilitation while in hospital)	7	Y
Permanent ventricular assist device	5	Y
New onset dementia	7	Y
New onset dialysis	4	Y
Limb amputation	3	N

© 2021 Sun LY et al. *JAMA Network Open.*

eTable 2. Description of Patient Centered Outcome Results: Questionnaire Round 2

Outcome	Value given to outcome	Included in next iteration (Y/N)
Any stroke regardless of severity	11	Y
≥3 non-elective hospitalizations per year	7	Y
Nursing home admission	10	Y
Single prolonged episode of hospitalization (≥40 days)	7	Y
Severe stroke (hospitalization ≥14 days or needing rehabilitation while in hospital)	12	Y
Permanent ventricular assist device	9	Y
New onset dementia	12	Y
New onset dialysis	8	Y
Heart failure (worsening heart function requiring hospitalization)	11	Y
Myocardial infarction	9	Y
Need for heart transplant	9	Y
Atrial fibrillation	5	N
Tracheostomy and/or ventilator dependence	7	Y
Immobility/paralysis	6	N
Inability to eat (dependence on feeding tube)	6	N
Death	5	N

© 2021 Sun LY et al. *JAMA Network Open.*

eTable 3. Description of Patient Centered Outcome Results: Questionnaire Round 3

Outcome	Value given to outcome	Included in next iteration (Y/N)
Any stroke regardless of severity	9	Y
≥3 non-elective hospitalizations per year	6	Y
Nursing home admission	9	Y
Single prolonged episode of hospitalization (≥40 days)	6	Y
Severe stroke (hospitalization ≥14 days or needing rehabilitation while in hospital)	11	Y
Need for a heart transplant or permanent ventricular assist device	8	Y
New onset dementia	10	Y
New onset dialysis	6	Y
Heart failure/ worsening heart function requiring hospitalization	8	Y
Myocardial infarction	7	Y
Tracheostomy	5	N
Ventilator dependence	6	Y
ICD	3	N

© 2021 Sun LY et al. *JAMA Network Open.*

eTable 4. Description of Patient Centered Outcome Results: Questionnaire Round 4

Outcome	Value given to outcome	Included for panel meeting (Y/N)
Any stroke regardless of severity	7	Y
≥3 non-elective hospitalizations per year	4	Y
Nursing home admission	7	Y
Single prolonged episode of hospitalization (≥40 days)	4	Y
Severe stroke (hospitalization ≥14 days or needing rehabilitation while in hospital)	9	Y
Need for a heart transplant or permanent ventricular assist device	6	Y
New onset dementia	8	Y
New onset dialysis	4	Y
Heart failure/ worsening heart function requiring hospitalization	6	Y
Myocardial infarction	5	Y
Ventilator dependence	5	Y

© 2021 Sun LY et al. *JAMA Network Open.*
